# Laboratory-based in situ and operando tricolor x-ray photoelectron spectroscopy

**DOI:** 10.1126/sciadv.adw6673

**Published:** 2025-08-22

**Authors:** Iris C. G. van den Bosch, Jahid Uz Zaman, Genrikh Shterk, Mai Hussein Hamed, Michael Schneider, Vadim Ratovskii, Yibin Bu, Paul M. Dietrich, Gertjan Koster, Christoph Baeumer

**Affiliations:** ^1^ MESA+ Institute for Nanotechnology, Faculty of Science and Technology, University of Twente, Enschede, Netherlands.; ^2^ Faculty of Science, Helwan University, Cairo, Egypt.; ^3^ SPECS Surface Nano Analysis GmbH, Berlin, Germany.

## Abstract

Innovative approaches to study buried interfaces and heterogeneous interactions under reaction conditions are crucial for advancing energy and catalytic materials. Our near-ambient pressure x-ray photoelectron spectroscopy (NAP-XPS) setup is equipped with a tricolor x-ray source, with Al Kα, Ag Lα, and Cr Kα excitation energies, enabling information depth–selective operando and in situ analysis of solid-liquid, solid-gas, and solid-solid interfaces. We present three case studies to demonstrate the systems’ capabilities. First, we compare experimental depth profiling of a LaMnO_3_/LaFeO_3_/Nb:SrTiO_3_ multilayer with SESSA (simulation of electron spectra for surface analysis) simulations. Second, we examine the oxidation and reduction of Fe*_x_*O*_y_* as a function of environment and temperature. Last, the Pt/liquid electrolyte interface is examined, revealing surface oxidation in the absence of bulk oxidation. As our results confirm, the unique combination of a NAP-XPS with the tricolor x-ray source empowers laboratory-based in situ and operando XPS characterization of advanced materials under reaction conditions in a wide range of applications.

## Teaser

Lab-based tricolor XPS reveals chemical changes in gas or liquid environments as a function of depth, temperature, and potential.

## INTRODUCTION

The need for advanced materials is rapidly increasing in a variety of fields, such as energy, sustainable chemistry, biomedical applications, and brain-inspired and quantum computing. As materials design grows increasingly complex, conventional empirical approaches to optimize functionality become more challenging. Consequently, a deeper understanding of the material properties, especially under external stimuli, has become essential and advanced characterization techniques are required to achieve this. With x-ray photoelectron spectroscopy (XPS), a surface-sensitive technique, information about the chemical composition and electronic structure can be revealed (*1*, *2*), making it attractive for knowledge-driven materials optimization.

For energy material applications, including but not limited to electrocatalysis, batteries, and gas-phase heterogeneous catalysis, an understanding of the material under reaction conditions is required. This is especially true if the formation of intermediate phases during the reaction governs functionality or if partially reversible transitions exist, which would remain undetected in pre– and post–reaction characterizations. The reaction conditions of interest include the presence of gaseous or liquid environments and/or elevated temperatures, requiring in situ or operando XPS studies. Here, we will use the definition “in situ” for measurements under controlled conditions, e.g., in a reaction gas and/or at a selected temperature, and “operando” for spectroscopic characterization during reactions with the simultaneous measurement of the functionality, e.g., the catalytic activity.

Performing in situ and operando XPS is challenging due to the short inelastic mean free path of photoelectrons, which typically necessitates vacuum conditions. Liquid or gas environments require a special instrument and reaction chamber design, which led to the development of near-ambient pressure (NAP) XPS. In the 1970s, the first differential pumping stages between a gas atmosphere near the sample and an electron analyzer at ultrahigh vacuum (UHV) were developed by K. Siegbahn and H. Siegbahn (*3*). Through a combination of differential pumping stages (*4*) and electrostatic lenses (*5*), NAP-XPS has now matured as an analytical technique (*6*). Initially, these NAP-XPS systems were primarily used to study solid-gas interfaces (*7*). For example, catalytic oxidation of carbon monoxide, nitrogen monoxide, and hydrocarbons can be performed to study a diverse range of properties of heterogeneous catalysts, including active site composition, metal-support interactions, and the effects of promoters (*8*–*13*).

In 2013, meniscus XPS was introduced as a new approach to measure the solid-liquid interface by creating a thin, photoelectron-transparent liquid meniscus at the measurement location (*14*). This development was a breakthrough, now enabling investigation of electrochemical (EC) solid-liquid interfaces under operando conditions with an increasing number of dedicated instruments around the world (*15*). Applications include the identification of electrode composition and electronic structure, overpotential and surface potential, and reaction intermediates in EC water splitting (*16*), fuel cells (*17*), and EC CO_2_ reduction (*18*). Another example is the study of solid electrolyte interfaces (SEI) for batteries (*19*, *20*).

Although most breakthroughs for in situ and operando XPS have been achieved at synchrotron facilities (*21*), also lab-based instrumentation is emerging (*22*, *23*). One of the advantages synchrotrons can offer is tunable information depth with different x-ray energies, allowing for depth profiling and probing different interfaces. This enables characterization of, for example, buried interfaces in solid-state batteries (*24*) and spintronics (*25*). For meniscus XPS, variable x-ray energies are particularly important to achieve increased probing depth through the electrolyte layer using tender or hard x-rays (*14*, *26*) or to tune the information depth toward interface sensitivity with soft x-rays (*27*). However, a substantial limitation of synchrotron-centered in situ and operando experiments is the comparably slow feedback and improvement cycles, which result from the inherently limited and proposal-based measurement time. Therefore, the development of laboratory-based NAP-XPS systems with in situ and operando capability and multiple x-ray energies spanning from soft to tender and hard x-rays is imperative. With our newly installed MESA+ laboratory-based in situ and operando tricolor NAP-XPS system, we can bridge this gap. Our tricolor source consists of three different excitation energies: Al Kα at 1487 eV, Ag Lα at 2984 eV, and Cr Kα at 5414 eV.

Here, we will demonstrate the capabilities of our in situ and operando tricolor XPS, using several case studies for validation. First, we describe the instrument overview and showcase the performance of the tricolor source on a standard Ag sample in UHV. Then, we demonstrate the depth profiling capability using a heterostructure of 5-nm LaMnO_3_/5-nm LaFeO_3_/Nb:SrTiO_3_ as a model system. The performance is validated by comparison with simulation of electron spectra for surface analysis (SESSA) simulations. In the next case study, the oxidation and reduction of Fe*_x_*O*_y_* are shown by heating in oxidizing and reducing atmospheres. We confirm previous expectations about the substrate-film interaction through the depth-selective information from the tricolor source. Last, the EC oxidation and reduction of Pt are shown. We demonstrate that this is a surface reaction using the tricolor source as the relative spectral intensity of surface oxides is highest with the Al Kα excitation and lowest with the Cr Kα excitation. Overall, our experiments showcase the opportunities arising from laboratory-based in situ and operando XPS across a wide range of applications. We highlight the possibilities enabled by the integration of multiple x-ray energies, which facilitates information depth tuning in laboratory-based NAP-XPS systems.

## RESULTS

### Instrument overview and performance characterization

**Fig. 1.**
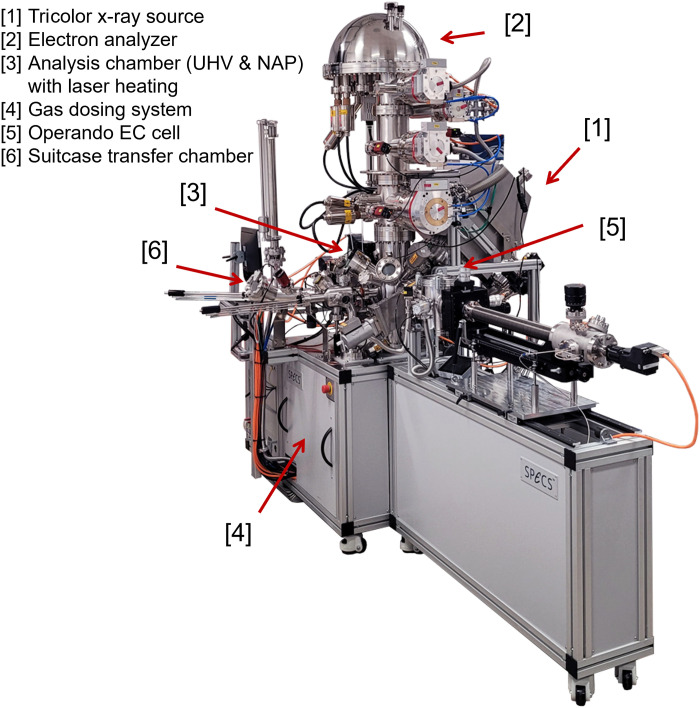
Overview of the tricolor NAP-system installed in the MESA+ Institute at the University of Twente. The instrument features a tricolor source, a vertically aligned analyzer, and two separate sample manipulators (back side: gas-solid manipulator with laser heating; front side: manipulator for the liquid EC cell).

A spectrometer’s geometry and electronic components considerably affect the scope of experiments. Figure 1 depicts the NAP-XPS system installed in the MESA+ Institute at the University of Twente. The combination of a vertically oriented analyzer, the tricolor x-ray source at magic angle, and the backfilling NAP-XPS instrument with a gas supply system enables various operando measurements of solid-gas and solid-liquid interfaces at a wide range of temperatures and gas pressures. In addition, it facilitates experiments with an open-type EC cell where the electrode is partially immersed in a liquid electrolyte. The NAP-XPS setup described in this work is based on the customized and modular FlexPS NAP Backfilling system by SPECS GmbH.

#### Tricolor monochromated x-ray source

**Fig. 2.**
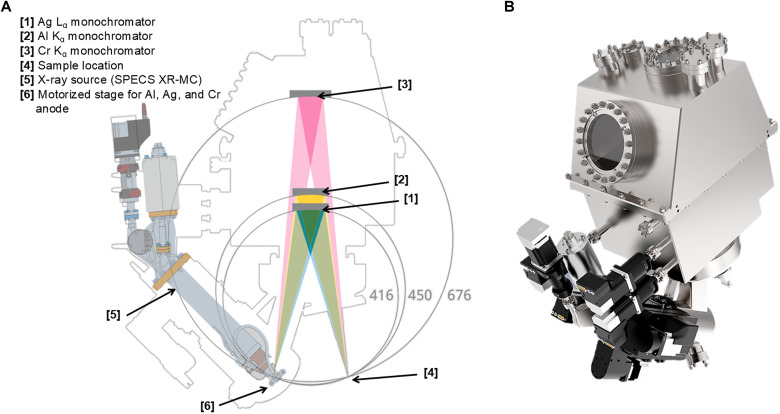
Schematics of the tricolor x-ray source. (**A**) Schematic cross section of the x-ray source. (**B**) Rendering of the x-ray source and monochromator compartment.

The μFOCUS 450 tricolor monochromated x-ray source is composed of a microfocus high-performance x-ray source (SPECS XR-MC) delivering variable spot sizes of less than 100 μm and more than 1 mm (Fig. 2 and Table 1). Photon flux values in Table 1 were measured using the μFOCUS 450 source with a phosphor screen and calibrated photodiode setup, accounting for diode responsivity and absorption by 6-μm aluminum foil. Spot size was determined from a two-dimensional (2D) Gaussian fit of the phosphor image on a glass substrate, yielding the 80 to 20% full width at half maximum (FWHM) as the effective size. The small spot source is equipped with Al, Ag, and Cr anode materials that can be exchanged in front of the electron gun via a motorized mechanism. The x-ray monochromator operates according to Bragg’s law of x-ray diffraction. Each wavelength of x-rays (Al Kα at 1487 eV, Ag Lα at 2984 eV, and Cr Kα at 5414 eV) is diffracted from individually optimized crystals at a specific angle of diffraction. The Al Kα and Ag Lα crystal mirrors can be moved in position and out of position with a motorized mechanism while the Cr Kα crystal mirror is stationary. This exchange of crystal mirrors in combination with selectable anode materials enables the fully computer-controlled switching of the three wavelength excitation energies, focusing each x-ray spot on the same sample position in the analysis chamber.

**Table 1.** Specifications of the tricolor monochromated x-ray source μFOCUS 450 Each row represents an anode material, and the columns indicate the spot size, flux and x-ray linewidth.

Anode material	Excitation energy (eV)	Spot size (μm)	Flux (photons/s), 250-μm spot	X-ray linewidth (FWHM) (meV)
Al	1487	100 to 1000	4.1 × 10^10^	220
Ag	2984	100 to 1000	1.2 × 10^9^	450
Cr	5414	200 to 1000	4.5 × 10^9^	500

For the Al and Ag anodes, quartz crystals are used for the x-ray mirrors, which have a 450- and 416-mm Rowland circle diameter, respectively, whereas for the Cr anode, the mirror is composed of germanium crystals with a 676-mm Rowland circle diameter. A Si_3_N_4_ window allows us to carry out XPS measurements under gas atmospheres of up to 25 mbar in the analysis chamber.

**Fig. 3.**
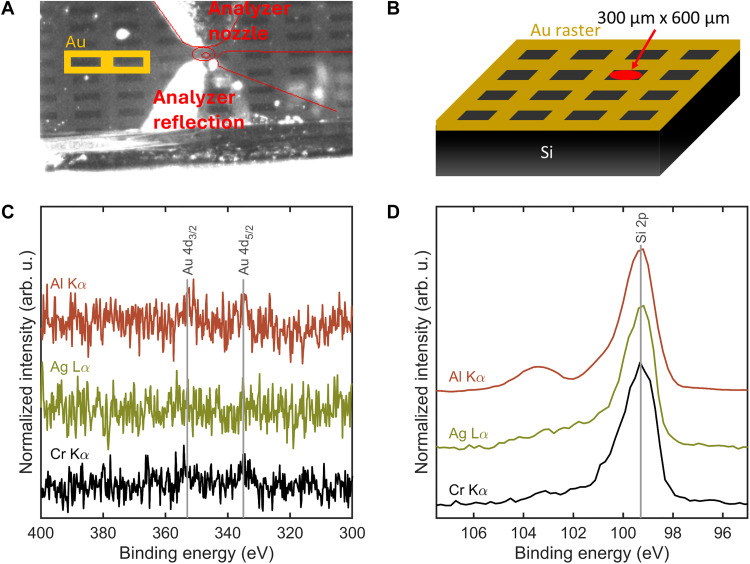
Demonstration of x-ray source switching in a confined measurement spot. (**A**) Camera picture of the nozzle and gold patterned silicon sample. (**B**) Schematic of the gold patterned silicon sample, the red dot between the gold grids indicating the measurement spot. (**C**) Au 4d spectrum, linear background subtracted and normalized. (**D**) Si 2p spectrum, Shirley background subtracted and normalized. Spectra were measured with a step of 0.2 eV, a dwell time of 0.2 s, a pass energy of 90 eV, and a nominal spot size of 100 μm. One, four, and eight scans are used of the respective Al Kα, Ag Lα, and Cr Kα excitations. arb. u., arbitrary units.

To demonstrate the switching of the x-ray source confined in one spot, measurements are conducted on a silicon sample with a gold grid on top (resulting in Si rectangles of 300 μm by 600 μm), as shown in Fig. 3 (A and B). With each excitation energy, Si 2p and Au 4d XP spectra are recorded, without moving the sample position between measurements. Figure 3 (C and D) shows no distinguishable Au peak from the noise and a strong Si peak, proving the confinement of the x-ray spot for all three excitations to be within 300 μm. No realignment of the source was needed for the different excitation energies. Movie S1 shows that the switching time from one excitation to the next takes about 1 min.

#### Analyzer

The hemispherical analyzer PHOIBOS 150 NAP is equipped with a 1D delay line detector (DLD), which provides a high count rate with minimal background noise. This detector also supports a wide photoelectron kinetic energy range up to 7 keV. The detector’s high reading speed allows measurement in “snapshot” mode; therefore, time resolution can be achieved in the case of high-intensity signal regions. The pre-lens and cone of the analyzer enable a wide acceptance angle of ±22°, ensuring efficient electron collection. It is connected to the analysis/NAP chamber by an electrostatic lens column containing three pumping stages, maintaining UHV conditions in the analyzer compartment even during NAP experiments. The pre-lens stage has a safety in-lens valve and a nozzle with an entrance diameter of 300 μm. This allows it to safely reach pressures up to 25 mbar with minimal risks of introducing high gas loads or liquid phase into the analyzer.

#### NAP chamber and manipulator

The NAP-XPS setup has two manipulators. A fully automated manipulator hosts the sample stage designed for cryogenic and high-temperature applications (150 to 1300 K) using liquid nitrogen cooling or infrared laser heating in different gas environments, as well as for routine UHV experiments. This stage can be operated remotely via the SpecsLab Prodigy software (*28*). The second manipulator, depicted on the right in Fig. 1, is the EC sample stage, as described in more detail below. A mass spectrometer MKS e-Vision 2 is installed in the second pumping stage to track the gas phase composition.

#### Gas dosing system

The analysis chamber functions as a UHV and NAP chamber due to the NAP backfilling system design. Two bypass valves with different diameters allow precise regulation of the gas phase pressure in the whole range, starting from UHV and up to NAP conditions (see fig. S1). The maximum pressure achievable in the setup is 25 mbar, limited by the electron mean free path in the gas phase. The gas dosing manifold consists of six gas lines, four of which are permanently connected (O_2_, CO_2_, H_2_O, and N_2_), whereas the remaining two are adjusted to user needs per experiment. All six gas lines are connected to the gas mixing manifold, from which the gas mixture is delivered into the NAP chamber. Degassed water vapor is created by bubbling He through Millipore water in a separately evacuable vessel, from which the water vapor is fed to a mass flow controller.

#### Operando EC cell

Although, nowadays, various EC cells ranging from simple dip-and-pull geometry to more complex flow cells with graphene-type photoelectron-transparent membranes allow the in situ or operando XPS investigation of the solid-liquid interface (*22*, *29*–*31*), we focus on meniscus XPS in the tilted sample geometry due to its versatile geometry, allowing facile sample design. Compared to the commonly used dip-and-pull geometry, the tilting configuration provides additional mechanical control over the meniscus formation, by changing the sample tilt. This is intended to enable gradual immersion/retraction of the liquid layer and the ability to tune the wetting behavior. The close-to-horizontal arrangement of the sample in meniscus XPS facilitates handling of the electrolyte and provides flexibility in the chamber and spectrometer design.

**Fig. 4.**
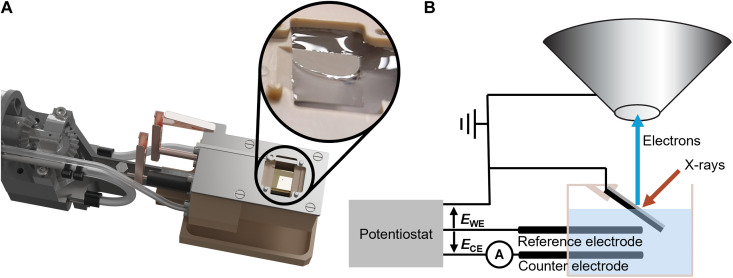
Liquid cell design. (**A**) Rendering of the design. Inset: Photo of a Pt thin-film sample partially immersed in an electrolyte. (**B**) Schematic of the tilted sample approach, including simplified electrical connections.

The EC cell features a windowless open-cell geometry where the sample or working electrode (WE) is fixed in the center, as shown in Fig. 4. Rotating the manipulator arm tilts the sample with respect to the analyzer and the liquid, allowing an adjustable immersion/submersion of the sample (WE) into the electrolyte. The reference electrode (RE) is a Ag/AgCl electrode, and the counter electrode (CE) is made of Pt wire. Such EC cell design allows the study of solid-liquid-gas interfaces on the edge of the electrolyte meniscus. In addition, the EC cell is designed to be in situ refillable, and precooled or heated electrolytes can be circulated inside the cell volume to ensure a stable meniscus, using two peristaltic pumps (internal and external). The electrolyte is degassed similarly to the water-vapor delivery system discussed above by using a separate chemically resistant membrane pump. In the future, membrane-type EC cells will be implemented into the system as well.

#### Suitcase

When handling air-sensitive samples and samples after synthesis or pretreatment in different facilities, it is crucial to preserve the native surface composition to perform accurate measurements. We use a vacuum suitcase, compatible with various UHV synthesis and characterization tools at the MESA+ Institute, across the Netherlands and beyond, to guarantee this. The vacuum suitcase consists of a vacuum cell (main body) equipped with a pressure gauge and battery-operational ion getter pump to ensure persistence of UHV conditions. In addition, it features a sample holder with screw thread for up to 10 flag-type sample plates and a gate valve with DN40CF size flange. A rendering of the suitcase is shown in fig. S2.

### Benchmarking with a Ag sample

To validate and demonstrate the capabilities of the tricolor x-ray source, measurements on a Ag sample were conducted such that the x-ray source can be benchmarked compared to other lab-based NAP-XPS instruments. A 5 mm–by–5 mm–by–0.2 mm Ag sample was cleaned in situ by oxidation of the surface at 2-mbar O_2_ at 620°C for 1 min, followed by a reduction at 2-mbar H_2_ at 620°C for 20 min. More commonly, sputtering is used to clean a sample surface, but this is not available on this tricolor NAP-XPS system. After this procedure, the surface consists of 84.5% Ag, 12.5% O, and 3% C, as quantified from a survey scan with Al Kα excitation.

**Fig. 5.**
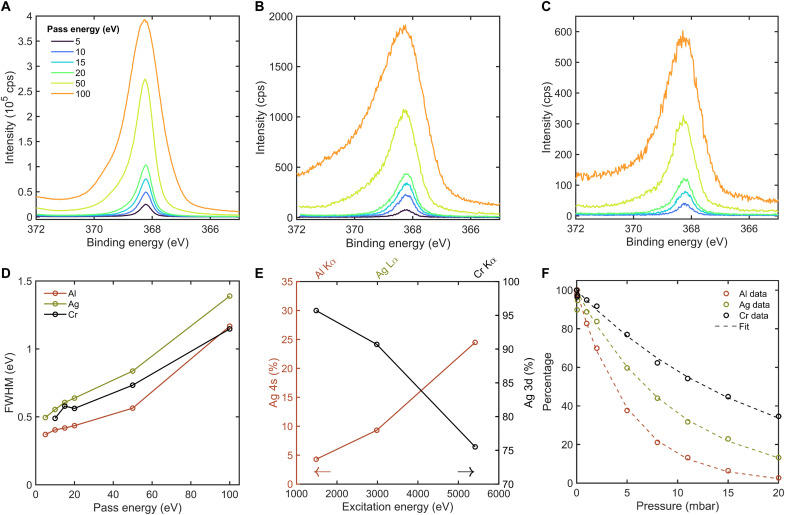
Benchmarking the tricolor source with a Ag sample. The Ag 3d_5/2_ peak of a Ag sample measured with different pass energies and with (**A**) Al Kα, with 0.02-eV step size, 0.2-s dwell time, and 1 scan; (**B**) Ag Lα, with 0.02-eV step size, 0.2-s dwell time, and 19 scans for 5- to 20-eV pass energy and 9 scans for 50- and 100-eV pass energy; (**C**) Cr Kα, with 0.02-eV step size, 0.2-s dwell time, and 25 scans for 10- to 20-eV pass energy and 16 and 9 scans for 50- and 100-eV pass energy. For all measurements, a Shirley background is subtracted. (**D**) FWHM of the three different excitation energies as a function of pass energy. (**E**) Integrated area percentage of the contributions of Ag 4s and Ag 3d5/2 as a function of excitation energy, measured with 0.05-eV step size, 100-eV pass energy, 0.2-s dwell time and one scan for Ag 3d5/2 and Ag 4s for Al Kα, 0.4- and 0.2-s dwell time and one and nine scans for Ag Lα, and 0.4-s dwell time and four and nine scans for the Cr Kα. (**F**) Area percentage of counts as a function of pressure, with UHV set to 100% and fitted with the Beer-Lambert law, measured with 0.05-eV step size, 100-eV pass energy, 0.2-s dwell time, and one scan for UHV to 8 mbar and two scans for 11 to 20 mbar. The sample was cleaned in situ by annealing in 2-mbar O2 followed by 2-mbar H2 at 620°C.

Figure 5 (A to C) shows the Ag 3d_5/2_ peak of the Ag sample measured with different pass energies with the tricolor source. It is apparent that the Al Kα gives the highest intensity, followed by the Ag Lα and the Cr Kα excitation. The FWHM shows that the Al Kα has the smallest linewidth and the Ag Lα has the largest natural linewidth (Fig. 5D, fig. S3).

The use of the tricolor source enables measurements of less accessible core levels as the photoionization cross sections of s, p, and d orbitals vary substantially with photon energy as discussed in numerous HAXPES studies (*32*). These trends can also be observed in our instrument. To demonstrate this, we compared the Ag 3d_5/2_ and Ag 4s core levels as a function of excitation lines. The area percentages of Ag 4s and Ag 3d_5/2_ are plotted as a function of excitation energy in Fig. 5E, with the corresponding raw spectra provided in fig. S4. The relative intensity of the Ag 4s (Ag 3d_5/2_) peak increases (decreases) when using higher excitation energies. This observation is consistent with the calculated cross sections reported by Trzhaskovskaya and Yarzhemsky (*33*). Specifically, the Ag 3d_5/2_ cross section decreases from ~130 to ~2.5 for photon energies ranging from 1500 to 5000 eV, whereas the Ag 4s cross section decreases only from ~7.8 to ~1.3.

To demonstrate the performance of the tricolor x-ray source at NAP conditions, the Ag 3d_5/2_ peak is measured as a function of different N_2_ pressures, as shown in Fig. 5F. The photoelectron intensity decreases most rapidly for Al Kα excitation, followed by Ag Lα and Cr Kα. This trend reflects the expected relationship between photoelectron kinetic energy and inelastic scattering probability: Electrons with higher kinetic energy are less attenuated by the gas phase.

The observed pressure-dependent intensity drop follows the Beer-Lambert law for photoelectrons, expressed as

$$\frac{I_{p}}{I_{0}}=e^{-z\cdot \sigma \left(\text{KE}\right)\cdot p/\mathrm{kT}}$$ (1)

where *I_p_* is the photoelectron peak intensity in the gas at pressure *p, I*_0_ is the photoelectron peak intensity in vacuum, *z* is the photoelectron travel distance through the gas, σ(KE) is the energy-dependent electron scattering cross section, *k* is the Boltzmann constant, and *T* is temperature (*34*). A value of σ(KE) = 0.99 × 10^−16^ cm^2^ for Al excitation was taken from literature data on total (elastic + inelastic) electron scattering cross sections in N_2_ gas (*35*). Using this value, we extracted a travel distance of *z* = 790 μm, which is consistent with the ~300-μm working distance plus the expected effective traveling distance in higher-pressure regions within the pumped analyzer nozzle. On the basis of this distance, the extracted cross-sectional values for Ag Lα and Cr Kα were 0.53 × 10^−16^ and 0.37 × 10^−16^ cm^2^, respectively. These are in reasonable agreement with the literature values of 0.37 × 10^−16^ cm^2^ (Ag Lα) and 0.22 × 10^−16^ cm^2^ (Cr Kα) (*36*), with the differences likely arising from geometry-dependent pressure gradients within the analyzer nozzle and additional photon attenuation in the gas phase between the Si_3_N_4_ window and the sample. At 20 mbar, the window-sample distance of 22 mm introduces an estimated photon absorption of ~5.5% for Al Kα, 0.7% for Ag Lα, and 0.1% for Cr Kα, based on calculations using Henke’s optical transmission database (*37*).

### Probing depth variation and solid-solid interfaces

**Fig. 6.**
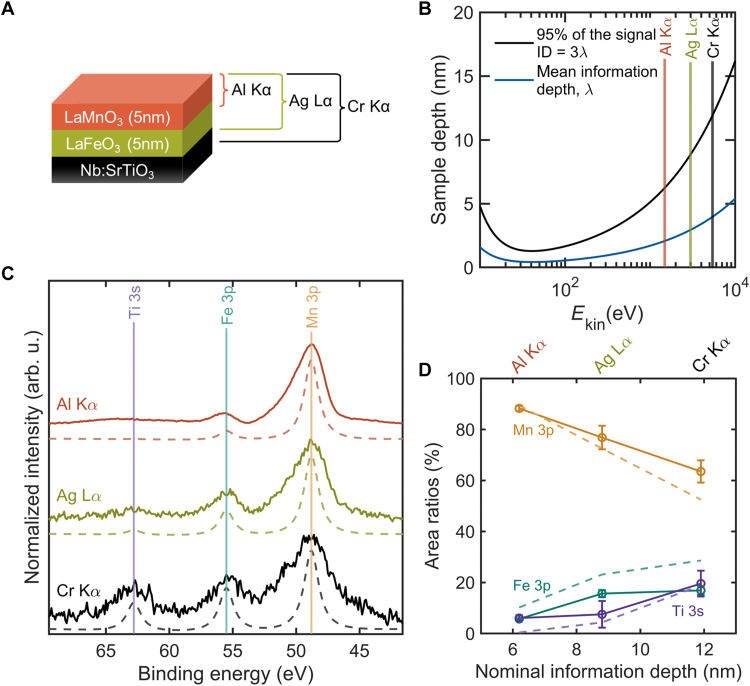
Depth profiling of 5-nm LaMnO_3_/5-nm LaFeO_3_/Nb:SrTiO_3_ solid-solid interfaces with the tricolor source. (**A**) Schematic representation of the heterostructure and available information depths using the tricolor source. (**B**) Universal curve for the mean information depth (1λ) and the 95% signal depth (3λ) (*46*), highlighting the information depth (ID) for the three different excitations. (**C**) Ti 3s, Fe 3p, and Mn 3p core-level spectra of the heterostructure sample, measured with different excitation energies. The measurements were conducted with a step size of 0.1 eV, a dwell time of 0.5 eV, a pass energy of 35, 50, and 100 eV, and number of scans of 20, 75, and 60 for the Al Kα, Ag Lα, and Cr Kα excitations, respectively. For analysis, the spectra were Shirley background subtracted, and the peak area ratios were calculated with CasaXPS. (**D**) Core-level area ratios of the sample as a function of nominal information depth for the experimental data and simulations. Solid lines and dashed lines in (C) and (D) represent the experimental data and the SESSA simulations, respectively. Error bars represent the SD in area integration in CasaXPS.

Solid-solid interfaces have always been at the focus of condensed matter research, with the pursuit of inducing and leveraging electronic and magnetic properties. Interfaces of even more complex materials can now be controlled, exploited, and interrogated at the atomic level. Epitaxial thin-film heterostructures (e.g., Fig. 6A) are a good example of such control (*38*), and such interfaces are at the foundation of many state-of-the-art and emerging technologies ranging from field effect transistors to ferroelectric tunnel junctions. Similarly, the solid-solid interface is gaining ever-increasing interest for (electro)chemical reactions, e.g., in corrosion (*39*), all solid-state batteries (*40*, *41*), and catalysis (*42*), for example, through so-called strong metal support interactions (*43*). In all these cases, the interfacial composition and (electronic) structure often deviate substantially from the bulk, which can both enable and impede desired functionality, requiring probing depth–selective characterization.

The XPS information depth depends on the photoelectron kinetic energies, where higher kinetic energies resulting from higher incident photon energies enable electrons to escape from deeper layers inside the sample without inelastic scattering. According to the Beer-Lambert law, the intensity *I* of electrons emitted from a buried layer decreases exponentially with increasing distance to the sample surface *d*

$$I=I_{o}e^{-\frac{d}{\lambda }}$$ (2)

where *I*_0_ is the total generated photoelectron intensity and λ is the inelastic mean free path. Approximately 95% of the electrons are emitted from a depth of *d* ≈ 3λ, corresponding to the XPS information depth according to ISO standards (*44*). The absolute λ values are tabulated in the NIST (National Institute of Standards and Technology) databases and can be predicted using the so-called TPP-2M formalism (*45*). For most materials, λ roughly follows a “universal curve” (Fig. 6B) (*46*). Although the exact inelastic mean free path values vary from material to material, the universal curve provides an instructive general estimate of the information depth as a function of electron kinetic energies. For shallow core levels like transition metal 3s and 3p peaks (binding energy of less than 100 eV), the resulting probing depth is ~7 nm for Al Kα, ~9 nm for Ag Lα, and ~13 nm for Cr Kα. Accordingly, variable energy XPS enables lab-based, nondestructive depth profiling of surfaces, subsurfaces, and buried interfaces regarding the electronic structure and chemical composition. Previously, such analyses were mostly available at large-scale x-ray facilities with tunable x-ray energy or in UHV-based laboratory XPS/HAXPES systems. Now, we can perform information depth–selective laboratory-based XPS also in the presence of gas and liquid environments.

To validate the depth profiling capabilities of our NAP-XPS system, we applied the tricolor x-ray source to a multilayered, epitaxial complex oxide heterostructure of 5-nm LaMnO_3_/5-nm LaFeO_3_/Nb:SrTiO_3_ as shown in the schematic representation in Fig. 6A. The heterostructure was grown using pulsed laser deposition (PLD); for details, see Materials and Methods and fig. S5. The maturity of epitaxy-based thin-film growth techniques provides precise control over layer and interface properties. Here, the epitaxy approach ensures unit-cell level thickness control, uniform layer thickness, and smooth surface topography, providing a suitable model system to assess the depth profiling capability.

SESSA simulations of the relative spectral contributions of each individual layer were performed to verify the experimental observations and compare them with quantitative expectations (*47*). This software package allows simulation of XP spectra for multilayered and structured thin-film samples using an NIST database. Details of simulation parameters are given in the Materials and Methods section.

We focus on the Mn 3p, Fe 3p, and Ti 3s core levels to demonstrate the tunable information depth of the tricolor source through the different layers of the heterostructures as shown in Fig. 6C (other core-level XP spectra are shown in fig. S7). We note that the photoionization cross section for all of these peaks is comparably low. We selected these peaks to compare separate peaks from each layer with nearly identical electron kinetic energy and to assess the capability in nonideal situations regarding the overall signal intensity.

As expected, and as predicted by the SESSA simulations, the intensity ratios between the core-level peaks vary depending on the excitation lines used. With a probing depth of ~7 nm for the Al Kα excitation, ~89% of the signal should originate from the 5-nm LaMnO_3_ top layer, according to Eq. 2. Experimentally, this expectation is confirmed by the relative intensity of Mn 3p (~90%) with only ~6% Fe 3p intensity, as shown in Fig. 6D. Virtually no Ti 3s peak is observed. When using the Ag Lα excitation, the probing depth extends further, reaching both the LaMnO_3_ and LaFeO_3_ layers and partially probing the buried Nb:SrTiO_3_ substrate. Therefore, the Fe 3p core-level peak intensity increases compared to the Al excitation, whereas the relative Mn 3p peak intensity decreases and a weak Ti 3s peak also appears. Last, with the Cr Kα excitation, besides the two films, a notable amount of the Nb:SrTiO_3_ substrate is probed, resulting in an increased relative intensity of Ti 3s peak. We note that, generally, s, p, and d orbitals exhibit different photoionization cross-sectional dependencies on photon energy, with s orbitals typically having a higher relative cross section at harder x-ray energies. This behavior is also confirmed in our experimental data for the Ag reference sample discussed above in Fig. 5E. For the Mn 3p, Fe 3p, and Ti 3s core levels, the Ti 3s level is expected to be more intense as the photoionization cross section for s orbitals at Cr Kα excitation is approximately twice as high as at Al Kα, whereas the enhancement for p orbitals is substantially lower (*33*). However, the variation in probing depth is also confirmed when analyzing the 2p orbitals of each layer (fig. S7), for which the cross-sectional dependence on photon energy is comparatively minor (*33*).

These observations are consistent with the results of SESSA simulation represented by the dashed lines in Fig. 6 (C and D). The qualitative trend between experimental observation and simulation results confirms the information depth tunability of the tricolor source. Small quantitative deviations may stem from interfacial and surface roughnesses and intermixing between layers, which are not captured in the simulation. Also, some additional uncertainties originating from the used inelastic mean free path calculations may contribute to these deviations.

### Temperature- and pressure-dependent chemistry

Understanding how the different layers in a complex heterostructure interact with the gas environment at solid-gas interfaces and with each other at the solid-solid interface is crucial for a range of applications, e.g., in heterogeneous catalysis, gas capture, and medicine (*25*, *48*–*52*). However, despite their importance, our understanding of the chemical composition at buried interfaces as a function of the surrounding gas atmosphere remains limited. Chemical- and depth-selective experiments are necessary to study reactions at both solid-solid and solid-gas interfaces to address this gap. The synchrotron-based XPS study of Fe*_x_*O*_y_* by Hamed *et al.* provides an example where the substrate, i.e., the buried layer, influences reactions at the gas-solid interface (*25*). In this section, we validate the functionality of our laboratory-based tricolor x-ray source to detect compositional changes at both solid-solid and solid-gas interfaces in the presence of different gas environments and at a range of temperatures.

**Fig. 7.**
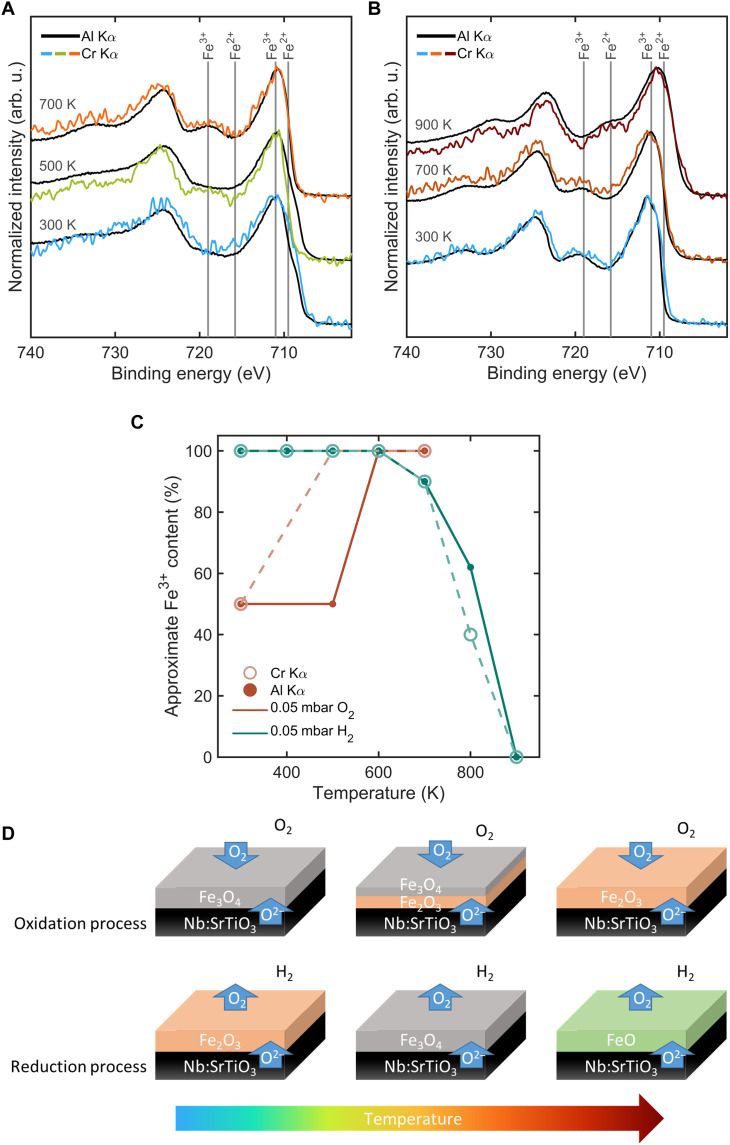
In situ depth profiling of Fe*_x_*O*_y_* during oxidation and reduction using tricolor XPS. (**A**) Fe 2p core-level spectra at 300, 500, and 700 K under 0.05-mbar O_2_, measured using both Al Kα and Cr Kα excitation. (**B**) Fe 2p spectra under 0.05-mbar H_2_ at 300, 700, and 900 K, measured using both Al Kα and Cr Kα excitation. The Cr Kα excitation data are smoothened with a quadratic Savitzky-Golay filter over eight data points. (**C**) Approximate Fe^3+^ content versus temperature extracted from linear combination fitting with FeO and Fe_2_O_3_ references. (**D**) Schematics of iron oxide thin films on SrTiO_3_ substrates and the oxidation-reduction processes of iron oxide phases driven by oxygen exchange across the solid-gas and solid-solid interfaces. The measurements were conducted with a step size of 0.1 eV and a dwell time of 0.5 s. For the Al Kα excitation, four scans were performed with a pass energy of 20 eV, whereas for the Cr Kα excitation, nine scans were performed with a pass energy of 50 eV.

Compositional variations and phase transitions and resulting tunable ranges of electronic and magnetic properties make iron oxides interesting materials for different applications in catalytic applications (*53*), spin-based technologies (*54*–*56*), and biotechnology (*57*). The three main phases of iron oxides are FeO (Fe^2+^), Fe_3_O_4_ (mixed Fe^2+^ and Fe^3+^), and Fe_2_O_3_ (Fe^3+^). The objective of this study is to track these phase transitions in situ under different conditions and probe the chemical composition of the surface (the solid-gas interface) and the subsurface (near the solid-solid interface). We measured the Fe 2p core-level spectra of a Fe_3_O_4−δ_ thin film grown on a TiO_2_-terminated Nb:SrTiO_3_ substrate by PLD under varying annealing temperatures and gas environments (oxygen and hydrogen) to monitor oxidation and reduction processes, inspired by the prior synchrotron study (Fig. 7, A to D) (*25*).

First, to monitor the oxidation process, the sample was annealed stepwise up to 700 K (see fig. S8A) under 0.05-mbar oxygen, with XPS measurements taken in situ using Al Kα and Cr Kα x-ray excitations. The Al Kα probes the surface, whereas the Cr Kα provides information about the subsurface and buried interfaces, as discussed above. Figure 7 shows the in situ depth-resolved Fe 2p core-level spectra of Fe*_x_*O*_y_* thin films during oxidation and reduction, measured using both Al Kα and Cr Kα excitation sources. Figure 7A presents the spectra recorded in 0.05-mbar O_2_ at 300, 500, and 700 K. At 300 K, the Fe 2p_3/2_ spectra exhibit features consistent with a mixed-valence Fe_3_O_4−δ_ phase (*58*), including a Fe^3+^ peak component near 711 eV and a shoulder at ~709 eV attributed to Fe^2+^. In addition, the satellite peaks at *E_B_* = 715.8 eV (Fe^2+^) and *E_B_* = 719 eV (Fe^3+^) are not apparent due to nearly equal contributions from Fe^2+^ and Fe^3+^. As the temperature increases, the Fe^2+^ shoulder diminishes, and by 700 K, it disappears, whereas a satellite peak at 719 eV becomes visible, indicating the formation of Fe_2_O_3_ (*58*). At 500 K, the Fe^3+^ satellite peaks are more evident with Cr Kα excitation than with Al Kα, confirming that the formation of the Fe_2_O_3_ phase begins at the buried interfaces. This indicates that the surface remains in the Fe_3_O_4−δ_ phase whereas the interface transitions to Fe_2_O_3_, as shown schematically in Fig. 7D. Thus, our experiments confirm the previous findings that the SrTiO_3_ substrate supplies oxygen via interfacial diffusion, causing Fe*_x_*O*_y_* oxidation near the substrate interface (*25*). These results highlight the importance of our tricolor XPS system for probing surface and interface layers under in situ conditions.

Second, we also investigated the subsequent reduction of the oxidized sample by annealing from 300 to 900 K with 100-K step in a 0.05-mbar hydrogen atmosphere (see fig. S8B). The XP spectra, shown in Fig. 7B, were measured using both Al Kα and Cr Kα excitation energies. At 700 K, the sample underwent partial reduction from Fe_2_O_3_ to Fe_3_O_4−δ_, as shown by the reappearance of the Fe^2+^ shoulder in the Fe 2p_3/2_ peak and the reduced intensity of the Fe^3+^ satellite peaks. At 900 K, further reduction to FeO*_y_* occurred, indicated by the disappearance of the Fe^3+^ satellite peaks and the emergence of Fe^2+^ satellite peaks (*58*). These findings indicate that the substrate- and atmosphere-induced phase transitions are reversible.

Figure 7C displays the approximate Fe^3+^ content as a function of temperature for both excitation sources and environments, obtained by linear combination fitting of reference spectra from FeO and Fe_2_O_3_ (see fig. S9). These values qualitatively reflect redox trends but are considered semiquantitative as fitting accuracy can be affected by background variability, especially at lower temperatures.

To further benchmark our tricolor system’s performance, we include a side-by-side comparison of Fe 2p spectra from our laboratory measurements and synchrotron-based data from Hamed *et al.* (*25*) in the Supplementary Materials (see fig. S10). Although such comparisons are inherently complicated by differences in experimental conditions, the qualitative agreement in peak shape confirms that the tricolor setup is capable of capturing the same phase transitions and redox behavior as synchrotron systems, albeit with the expected lower count rate.

Overall, our in situ ambient-pressure XPS measurements with the variable information depth resulting from Al Kα and Cr Kα x-ray excitations successfully monitored the oxidation and reduction of iron oxide surfaces and subsurfaces under controlled temperature and pressure conditions. This capability enables precise analysis of phase transitions and chemical changes at solid-solid and solid-gas interfaces in complex systems, providing valuable insights into catalysis and related applications.

### Liquid electrochemistry

The liquid EC cell enables operando characterization of electrocatalysts and other EC materials. One of the most studied materials in operando XPS is platinum (*22*, *59*–*62*). Pt is a widely used catalyst for the hydrogen evolution reaction (HER) in water electrolyzers and for hydrogen oxidation and oxygen reduction in fuel cells, in both alkaline and acidic media. A better understanding of the behavior under reaction conditions is required to limit the platinum consumption, especially regarding platinum oxidation and subsequent platinum oxide reduction processes, which are known to induce dissolution (*63*). In this section, a case study of oxidation and reduction of a Pt electrocatalyst is discussed, using the EC cell described in the section Instrument overview and performance characterization. In addition, to minimize electrolyte evaporation, the electrolyte is precooled to ~13°C, and a 25-mbar H_2_O chamber pressure was used. The tricolor source is used to verify previous estimates and expectations that PtO*_x_* forms mostly at the surface and to validate our capability of information depth–selective operando XPS.

**Fig. 8.**
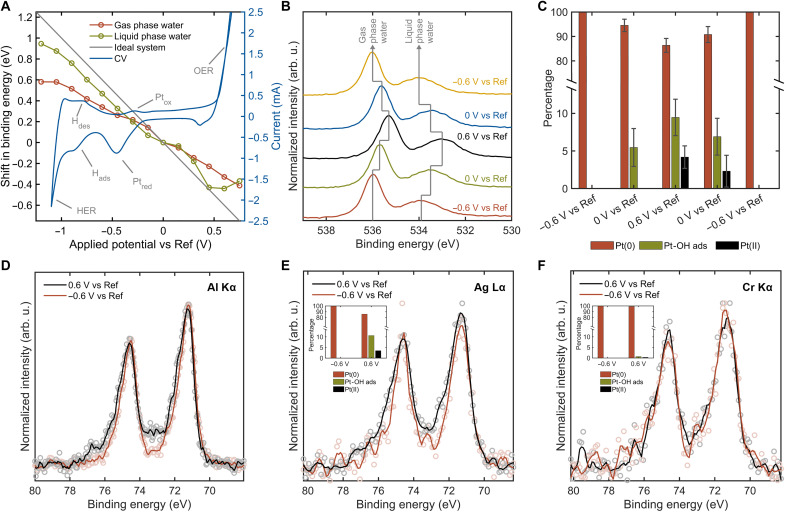
Operando tricolor XPS of the Pt/electrolyte interface. XP spectra of Pt 4f and O 1s regions excited with Al Kα [(A) to (D)], Ag Lα (E), and Cr Kα (F) photons during an experimental run on a 50-nm Pt thin-film WE. (**A**) XPS peak shifts versus applied potential overlaid with cyclic voltammogram. OER, oxygen evolution reaction. (**B**) O 1s peaks as a function of applied potential, Shirley background subtracted and normalized. Spectra were recorded using a step size of 0.1 eV, dwell time of 0.5 s, pass energy of 20 eV, and three scans per potential. (**C**) Relative contribution of various Pt oxidation states obtained from fits. Reversible Pt oxidation and reduction are apparent from the potential-determined peak contribution of metallic and oxidic species. Monte Carlo simulations built into CasaXPS were used to estimate the error bars based on the uncertainties in peak area resulting from random noise in the data. (**D**) Pt 4f peaks as a function of applied potential, Shirley background subtracted and normalized. Spectra were recorded using a step size of 0.05 eV, dwell time of 1 s, pass energy of 20 eV, and three scans per potential. XPS of Pt 4f core levels at −0.6 and 0.6 V versus Ref measured with (**E**) Ag Lα and (**F**) Cr Kα excitation. Spectra were acquired with a 0.1-eV step size, 2-s dwell time, and pass energies of 50 eV (Ag Lα) and 100 eV (Cr Kα). The number of scans was 11 and 7 for Ag Lα and 8 and 12 for Cr Kα at −0.6 and 0.6 V versus Ref, respectively. Insets show the relative contributions of different Pt oxidation states from peak fitting analysis.

Figure 8A shows a typical cyclic voltammogram for Pt versus a Ag/AgCl RE, further referred to as versus Ref. The cyclic voltammogram shows the expected signatures of oxidation and reduction peaks of the Pt, the oxygen evolution reaction, the adsorption and desorption of hydrogen, and the HER.

For operando characterization, it is crucial to confirm the actual applied potential at the measurement location, which may deviate from the potentiostat set point due to mass transfer limitations in the thin meniscus. This is readily achievable by tracking the binding energy position of electrolyte XPS peaks. As the sample and analyzer are connected to the same ground, the WE XPS signal is solely governed by chemical changes, implying no direct potential-dependent shift of the XPS peaks. In contrast, because the EC potential is applied to the liquid (with respect to the RE), all XPS levels of the electrolyte exhibit a so-called rigid shift toward lower binding energies for positive applied potentials and higher binding energies for negative potentials (*64*). In the absence of ohmic losses, the rigid shift and applied potential differences follow a 1-eV/1-V trend.

In Fig. 8B, the O 1s spectra measured with the Al Kα excitation are shown as a function of applied potential, where two distinct features can be noticed: The gas and liquid phase water peaks. As expected, applying oxidizing (reducing) potentials shifts the spectra toward lower (higher) binding energies. In Fig. 8A, the O 1s peak shifts of gas and liquid phase water almost coalign with the ideally expected trend, where the deviation is caused by a potential drop due to higher resistance of the thin meniscus. An additional cause of the deviation is a possibly inhomogeneous electrolyte meniscus, where parts of the measurement area do not contribute to the expected shift (*65*). The observed shift in the gas phase is lower than for the liquid phase, caused by an additional potential drop within the gas phase (*66*).

Figure 8 (C and D) shows the Pt 4f spectra and contribution of different Pt oxidation states as a function of potential. Figure 8D shows the Pt 4f XPS peaks at positive and negative potentials versus Ref, measured with the Al Kα excitation. We observe oxidation at positive potentials and a reduction at negative potentials. The Pt 4f spectra were recorded at sequentially applied potentials of −0.6, 0, 0.6, 0, and −0.6 V versus Ref (see fig. S11). These peaks were fitted with CasaXPS using a peak model with Pt(0), Pt-OH adsorbates, and Pt(II) at 71.0, 71.9, and 73.5 eV, respectively (a detailed description can be found in fig. S12). In Fig. 8C, the percentage of the different peaks is shown as a function of applied potential. Here, the pristine sample is fitted with 100% Pt(0) at −0.6 V versus Ref. Once we start applying oxidizing potentials at 0 V versus Ref, the amount of Pt(0) decreases to 94.5% and OH adsorbates start to form on the surface resulting in 5.5% Pt-OH. At the highest oxidizing potential used, just at the onset of the oxygen evolution reaction at 0.6 V versus Ref, a further decrease in Pt(0) to 86.4% and an increase in concentration of Pt-OH to 9.5% is observed. In addition, we start forming 4.2% Pt(II) at these potentials, corresponding to PtO. This oxidation is expected according to the Pourbaix diagram (*67*). Upon decreasing the potential to 0 V versus Ref, Pt(0) increases to 90.8% and Pt-OH and Pt(II) decrease to 6.9 and 2.3%, respectively. Last, back at −0.6 V versus Ref, the sample is fully reduced to its original condition.

Figure 8 (E and F) shows the Pt 4f XPS peaks at reducing and oxidizing potentials, measured with the Ag Lα and Cr Kα excitation, respectively. In the insets, a bar diagram shows the results of the same peak fit model used for the Al Kα excitation. With the Ag Lα excitation at −0.6 V versus Ref again the peak is fitted with 100% Pt(0), and at 0.6 V versus Ref, an oxidation is visible with 10.6 and 3.5% of Pt-OH and Pt(II), respectively, and a decrease in Pt(0) to 85.9%, overall similar to the measurements with Al Kα excitation. For the Cr Kα excitation, no significant oxidation is observed, with 99% Pt(0) and only 1% oxidized species. As discussed and confirmed in the section Probing depth variation and solid-solid interfaces, the Al Kα excitation is the most surface-sensitive technique, and once one starts to use harder x-rays, like the Ag Lα and Cr Kα excitations, spectra with higher mean information depth are measured. Therefore, we suggest that the EC oxidation and reduction are surface reactions. This is in agreement with the simulations conducted by Favaro *et al.* (*59*), who suggested potential-dependent Pt-OH and Pt oxide layers based on synchrotron-based XPS with a single x-ray energy.

## DISCUSSION

In summary, our laboratory-based tricolor NAP-XPS system demonstrated its versatility in studying buried interfaces and layers under in situ and operando conditions. Its ability to perform depth-selective XPS analysis of solid-solid, solid-gas, and solid-liquid systems has been validated through various case studies, and the extracted information revealed the opportunity for added insights from the application of the tricolor source to NAP-XPS.

Depth profiling of a LaMnO_3_/LaFeO_3_/Nb:SrTiO_3_ heterostructure system in UHV and Fe_3_O_4_ thin films under the influence of gas environments and temperature highlighted the systems’ ability in studying buried layers and in tracking oxidation-reduction transitions under a controlled atmosphere. Surface oxidation was observed using Al Kα, showing Fe^3+^ formation at 700 K, whereas more bulk-sensitive measurements with Cr Kα found the transition to Fe^3+^ already at 500 K, indicating deeper oxidation near the substrate interface. The operando liquid electrochemistry study demonstrated the systems’ potential to probe EC processes at the solid-liquid interface by investigating a platinum electrode under various potentials. While performing meniscus XPS in tilted sample geometry, the tricolor XPS effectively resolved oxidation and reduction of the Pt electrode, where the information depth tunability provides a substantial advancement compared to previous analyses. Surface-sensitive investigation with Al Kα excitation revealed more pronounced oxidation than with Cr Kα excitation, confirming that the oxidation and reduction are surface-dominated processes, with no substantial bulk oxidation observed by using harder x-rays. With the tricolor source, we are able to observe how a material changes as a function of reaction conditions and information depth. This multienergy approach enables a more nuanced understanding of catalytic processes. As emphasized in our previous work (*27*), such a development is crucial for addressing key challenges in electrocatalysis. The underlying rationale is that, although harder x-rays enhance the relative signal of the solid with respect to the liquid in meniscus XPS, softer x-rays preferentially enhance the signal from the solid surface over its bulk. Therefore, the combination of soft, tender, and hard x-rays provides a powerful means to deconvolute the depth-dependent changes in electrocatalysts under reaction conditions. This depth-resolved insight is instrumental for the rational design and optimization of functional materials.

The tricolor NAP-XPS system represents a notable advancement in interface analysis under reaction conditions, relevant for a large field of applications. This tool not only enhances the information depth selectivity and scope of interfacial studies but also opens pathways for addressing complex scientific challenges across multiple disciplines. In addition to classical applications in heterogeneous catalysis, our tool also allows to measure phase transitions as a function of temperature and pressure, e.g., metal to insulator transitions (*68*), and measurements under typical synthesis conditions of advanced synthesis techniques like PLD or molecular beam epitaxy, providing valuable insights into the material formation and transformations, especially in the presence of buried interfaces (*69*). Combining the tricolor approach with the designed EC cell with electrolyte supply and preconditioning fulfills a previously identified gap regarding the information depth selectivity and depth profiling of electrochemically induced changes on both sides of the solid-liquid interface and promises enhanced stability of the electrolyte meniscus (*23*, *27*). The tricolor meniscus XPS approach may also be valuable for investigating electric double layer effects. Although the present study focuses on high-concentration electrolytes (0.1 M KOH), which result in short Debye lengths and symmetric O 1s peak shapes, low-concentration electrolyte conditions, where the Debye length is longer, may allow binding energy shifts arising from interfacial potential gradients to become resolvable (*2*, *70*). We expect that the tricolor source would lead to different asymmetric broadening of the electrolyte XP spectra for different mean information depths, which would help in solidifying the extracted potential profiles.

Although the tricolor laboratory-based NAP-XPS system provides substantial versatility and depth resolution, it does have some limitations. The lower photon flux of the Cr and Ag excitations compared to Al combined with decreasing photoionization cross sections with increasing photon energy results in longer acquisition times and reduced signal-to-noise ratios. The surface sensitivity can be further enhanced in vacuum, gas, and liquid environments using grazing emission detection, but the tilt angles are limited to ~50°, in practice due to the short working distance between the sample and analyzer cone. In addition, the operando EC configuration still faces challenges in meniscus stability and reproducibility. Although these currently limit precision in interfacial potential mapping and experiment time, future developments including improved liquid handling and cell design may contribute to overcoming these challenges. We note that similar challenges are also present at synchrotron-based solid/liquid experiments and that access to a laboratory-based tool may optimize feedback and optimization cycles.

Future advancements in operando cell design will also benefit from the tricolor source, which allows information depth selection also for cells with photoelectron-transparent membranes. The coexistence of laboratory-based and synchrotron-based in situ and operando XPS systems creates possibilities in experimental workflows. Laboratory-based systems enable feasibility studies, assessment of x-ray effects, and iterative operando cell optimization even before synchrotron experiments begin, ensuring the efficient use of limited synchrotron beam time. This collaborative approach accelerates research progress by providing initial insights while reserving synchrotron resources for detailed, time-sensitive investigation, for example, for improved time-resolution and highly improved signal-to-noise ratios due to the high flux. A laboratory-centered approach also opens access to in situ and operando XPS to a larger scientific community.

Looking ahead, the tricolor XPS user facility at the MESA+ Institute will address some of the most pressing questions in materials science, providing an approach for fundamental research targeted toward practical applications in sustainable technology development.

## MATERIALS AND METHODS

### Sample preparations

The LaFeO_3_ and LaMnO_3_ heterostructure was grown using PLD (TSST custom-made at MESA+ Institute) on a TiO_2_-terminated Nb:SrTiO_3_ substrate, treatment procedure reported elsewhere (*71*). The KrF excimer laser (λ = 248 nm) with the spot size of 1.8 mm^2^ was used to ablate the LaFeO_3_ and LaMnO_3_ stoichiometric targets. During the deposition, a substrate-target distance of 50 mm, growth temperature of 670°C, background O_2_ partial pressure of 0.01 and 0.1 mbar, laser fluence of 1.8 and 1.85 J/cm^2^, and frequency of 2 and 1 Hz were used for LaFeO_3_ and LaMnO_3_ layers, respectively.

Epitaxial iron oxide (Fe*_x_*O*_y_*) films were grown in the same PLD setup with a background O_2_ pressure of 5 × 10^−6^ mbar, 450°C temperature, and KrF laser ablating the FeO ceramic target at a fluence of 1.5 J/cm^2^ and 5-Hz frequency. Reflective high electron energy diffraction was used to monitor the growth of the heterostructure with unit cell precision (see fig. S6).

The WE for liquid electrochemistry was prepared by depositing a 50-nm-thick Pt layer onto a silicon wafer substrate (10 mm by 10 mm) using dc sputtering. The silicon substrate was cleaned ultrasonically in acetone, ethanol, and deionized water sequentially before sputtering to ensure a clean and uniform surface.

### SESSA simulations

SESSA simulations were conducted to verify the comparison of the spectra with the three different excitation energies. For the simulations, a planar morphology was chosen. In Table 2, the simulated parameters are summarized. Binding energies in the SESSA database are given for metallic samples, and a range of applicable chemical shifts is tabulated for various oxidation states. Here, the chemical shifts were applied to the simulated spectra to match the binding energy of the experimental data.

**Table 2.** SESSA simulation settings for the 5nm LaMnO_3_/5nm LaFeO_3_/Nb:SrTiO_3_ heterostructure. The different rows represent the different layers, and the columns contain the simulation parameters.

Layer	Thickness (Å)	Density (g/cm^3^)	Valence electrons	Bandgap	Chemical shift (eV)	
SrTiO_3_	–	4.96	28	–	Ti 3s	4.1
LaFeO_3_	50	6.51	28	2.65	Fe 3p	2.8
LaMnO_3_	50	6.57	28	1.02	Mn 3p	1.6

### Temperature- and pressure-dependent chemistry

The sample was first loaded into the HAXPES chamber and degassed under UHV conditions at room temperature to identify the initial oxidation state of the sample by recording Fe 2p core levels. For the oxidation process, oxygen gas [O_2_, 99.9999% purity (Linde, Germany)] was introduced into the chamber at a pressure of 0.05 mbar. The sample temperature was increased stepwise from 300 to 700 K in increments of 100 K, and core-level spectra of Fe 2p were acquired at each temperature to monitor changes in oxidation state using first the Al Kα excitation. This was followed by measurements with the Cr Kα excitation. After completing the oxidation process, the chamber was evacuated to UHV and refilled with hydrogen gas [H_2_, 99.999% purity (Linde, Germany)] at a pressure of 0.05 mbar for the reduction process. The temperature was increased from 300 to 900 K with 100-K increments, and core-level spectra for Fe 2p were acquired at each temperature using both Al Kα and Cr Kα excitations in the same sequence. Stable chamber pressure was maintained throughout the experiment by proportional–integral–derivative controller settings of the pressure control system, ensuring consistent and reliable measurement conditions. This stepwise approach provided detailed insights into the thermal redox behavior of Fe*_x_*O*_y_* under varying atmospheric and thermal conditions.

### Liquid electrochemistry

For the liquid electrochemistry experiments, a three-electrode EC setup was used, consisting of a WE, a platinum CE, and a Ag/AgCl RE. The WE was the sputtered Pt sample on a Si wafer substrate. Before introducing the sample into the EC chamber, cyclic voltammetry was performed in a potential window of −1.1 to 0.9 V versus Ag/AgCl at a scan rate of 100 mV/s to observe Pt redox features. The electrolyte solution was 0.1 M KOH prepared using Millipore water. Before inserting the cell into the XPS chamber, the electrolyte was cooled to 13°C and stabilized at a pressure of 25 mbar. In addition, the analysis chamber pressure was set to 25-mbar water vapor. After transferring the cell into the chamber, Pt 4f and O 1s core-level spectra were measured using Al Kα excitation while applying a constant voltage sequence of −0.6, 0, 0.6, 0, and −0.6 V versus Ag/AgCl with the chronoamperometry method. For the Ag Lα and Cr Kα x-ray excitations, Pt 4f and O 1s core-level spectra were measured at −0.6 and 0.6 V versus Ag/AgCl only, due to the longer measurement times associated with these excitations. All EC measurements were carried out using a Biologic SP-200 potentiostat, and XPS data analysis was done using CasaXPS software.
