# Supplementary material for: Laboratory-based in situ and operando tricolor x-ray photoelectron spectroscopy
Source: Sci Adv. 2025 Aug 22;11(34):eadw6673. doi: 10.1126/sciadv.adw6673 (PMC12372890; doi:10.1126/sciadv.adw6673)
Supplement: Supplementary file 1 — Figs. S1 to S12 Table S1 Legend for movie S1 [file sciadv.adw6673_sm.pdf]

Science Advances
AAAS

Supplementary Materials for

Laboratory-based in situ and operando tricolor x-ray photoelectron spectroscopy

Iris C. G. van den Bosch *et al.*

Corresponding author: Christoph Baeumer, c.baeumer@utwente.nl

Sci. Adv. **11**, eadw6673 (2025)
DOI: 10.1126/sciadv.adw6673

The PDF file includes:

Figs. S1 to S12
Table S1
Legend for movie S1

Other Supplementary Material for this manuscript includes the following:

Movie S1

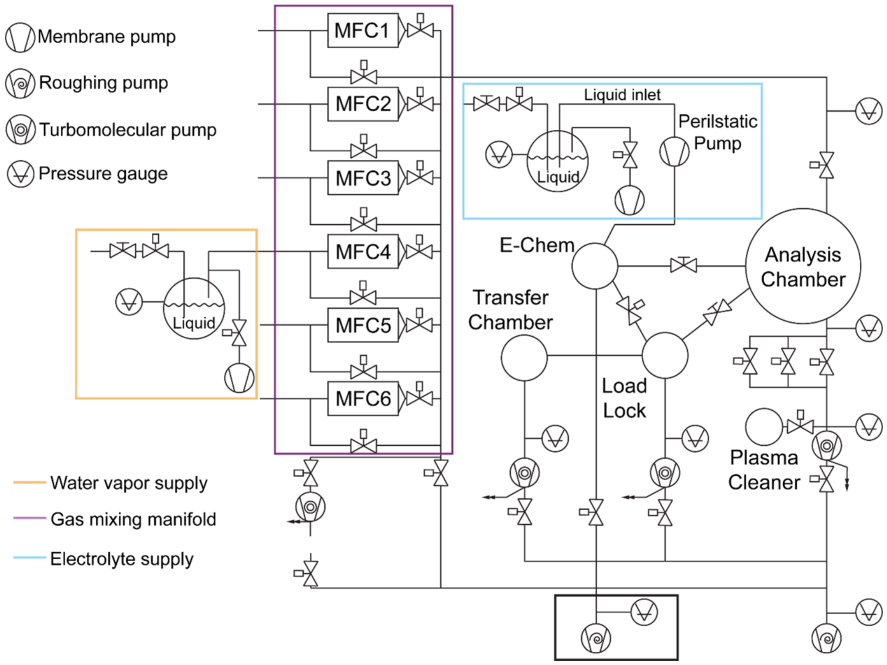

Figure S1: **Schematic of the gas dosing and vacuum system.** Shaded boxes indicate individual aspects, including the gas mixing manifold (violet), the water vapor supply (yellow) and the electrolyte handling in the electrochemical chamber (blue).

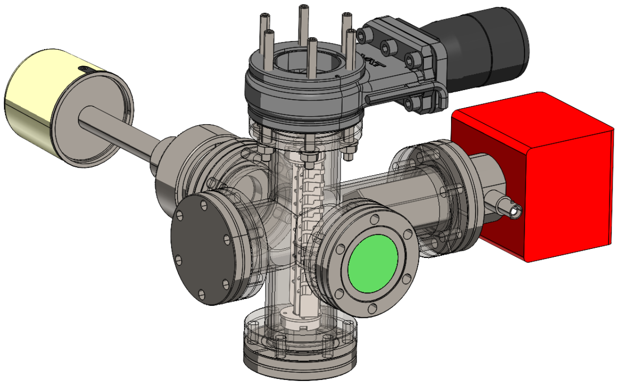

Figure S2: **Vacuum suitcase rendering.** On top the DN40CF size flange, on the left the pressure gauge, on the right the ion getter pump and in the middle a carousel with place for ten flag-type sample plates. Drawing: Courtesy of Dominic Post.

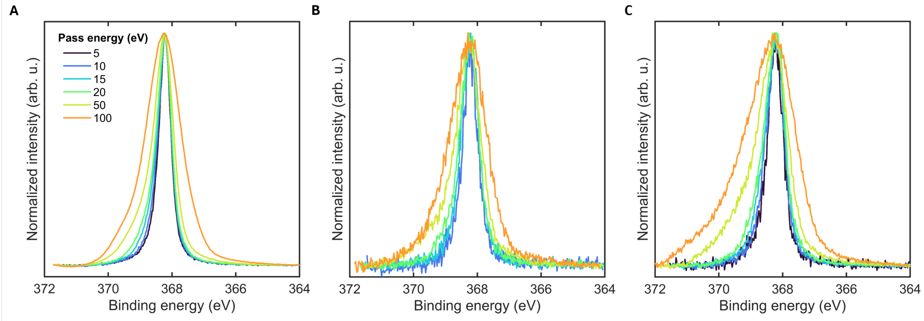

Figure S3: **Normalized XP spectra on Ag benchmarking sample.** Shirley-background-subtracted Ag 3d5/2 spectra measured with A) Al Kα, B) Ag Lα and C) Cr Kα excitation.

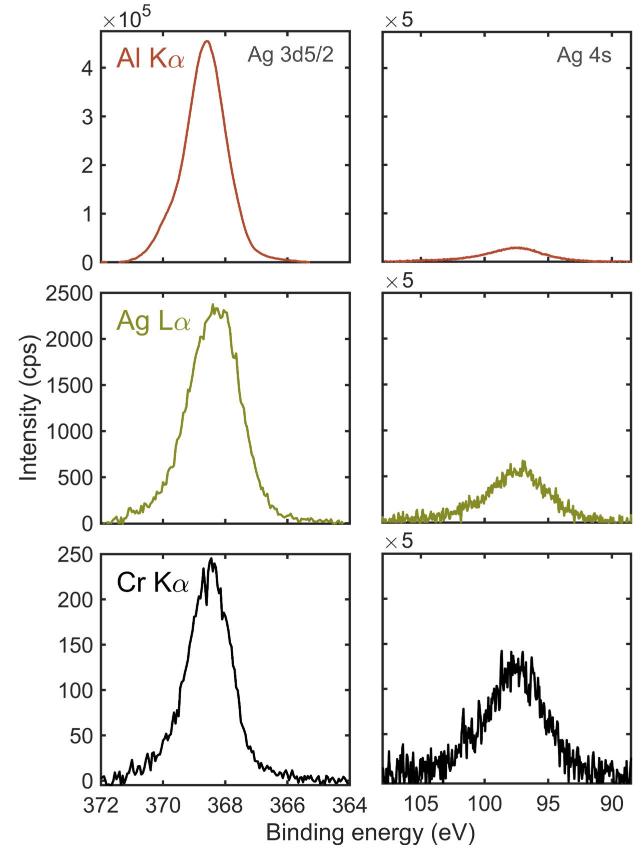

Figure S4: **XP spectra of Ag 3d5/2 and Ag 4s on Ag benchmarking sample measured with Al Kα, Ag Lα and Cr Kα excitation.**

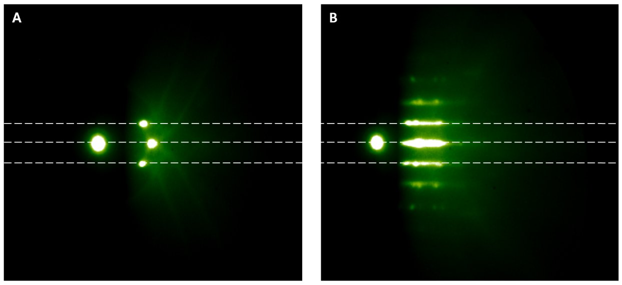

Figure S5: **RHEED pattern of 5 nm $LaMnO_3$/5 nm $LaFeO_3$/Nb:$SrTiO_3$.** A) NbSTO substrate before growth and B) after growth of $LaMnO_3$.

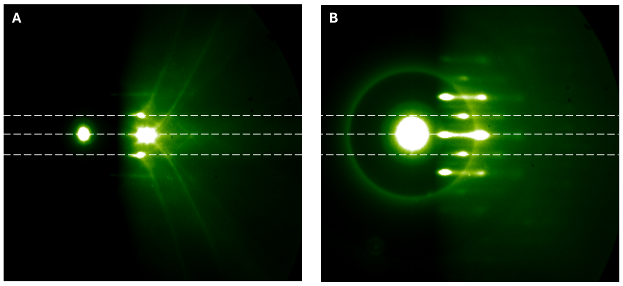

Figure S6: **RHEED pattern of Fe_xO_y/Nb:$SrTiO_3$.** A) NbSTO substrate before growth and B) after growth of Fe_xO_y.

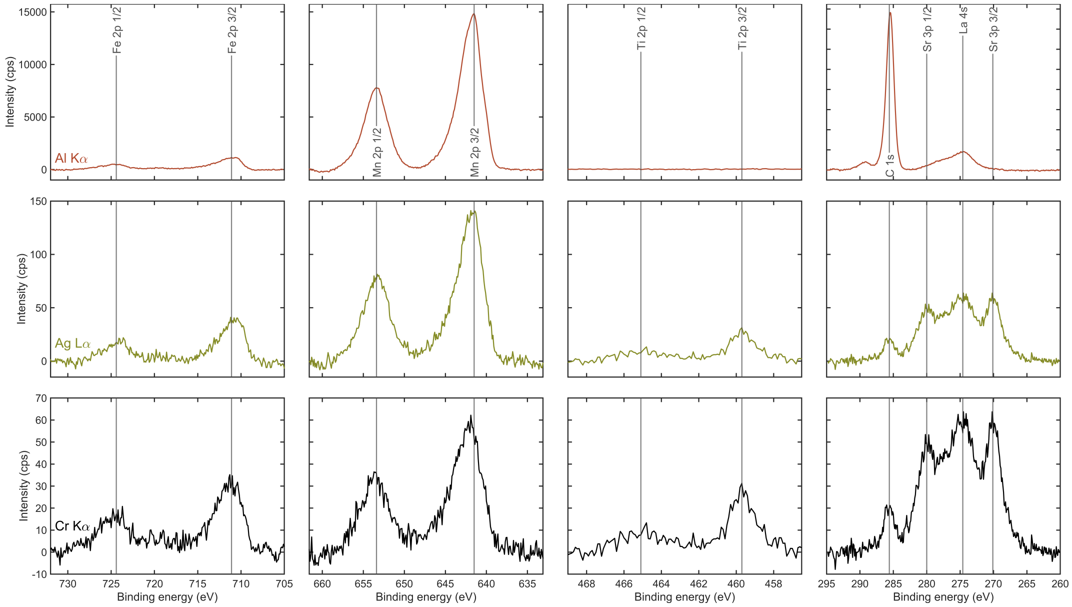

Figure S7: **The core level spectra of 5 nm $LaMnO_3$/5 nm $LaFeO_3$/Nb:$SrTiO_3$, probed with the tri-color source.** The Fe 2p, Mn 2p, Ti 2p, Sr 3p and La 4s core levels of the 5 nm $LaMnO_3$/5 nm $LaFeO_3$/Nb:$SrTiO_3$, Shirley background subtracted. Probed with the different excitations, from top to bottom Al K α, Ag L α and Cr K α. With the Al Kα excitation, the spectra are acquired with a step size of 0.1 eV, 35 eV pass energy and a dwell time of 0.5 s. 30, 10, 20 and 15 scans are used for the respective panels left to right. With the Ag Lα excitation, a step size of 0.1 eV, 50 eV pass energy and a dwell time of 0.5 s. 50, 30, 48 and 15 scans are used for the respective panels left to right. With the Cr Kα excitation, a step size of 0.1 eV, 100 eV pass energy and a dwell time of 0.5 s. 50, 30, 30 and 20 scans are used for the respective panels left to right.

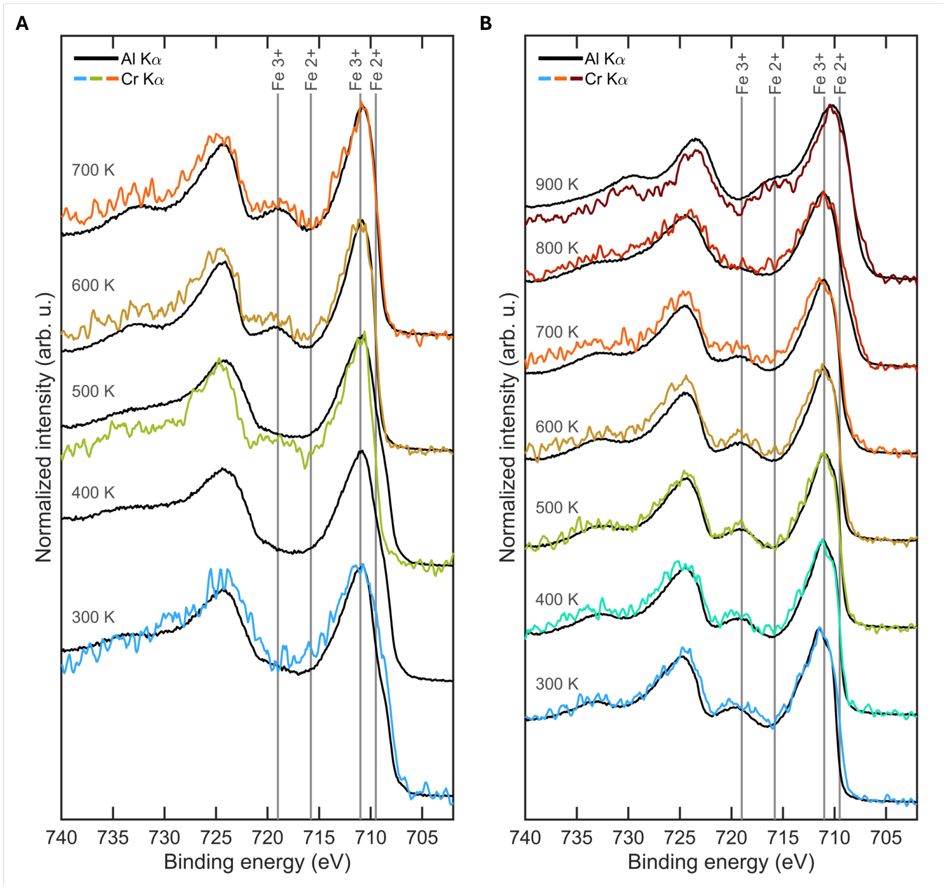

Figure S8: **Core-level spectra of Fe 2*p* in A) oxidizing and B) reducing conditions**, measured with Al Kα and Cr Kα. For Al Kα excitation, the spectra were acquired with a step size 0.1 eV, 20 eV pass energy and a dwell time 0.5 s with 4 scan recorded at each temperature steps. For Cr Kα excitation, the spectra were acquired with the same step size and dwell time but with a pass energy of 50 eV, recording 9 scans for each temperature step. For Cr Kα excitation, solid lines show smoothened data with a quadratic Savitzky-Golay filter over 8 data points.

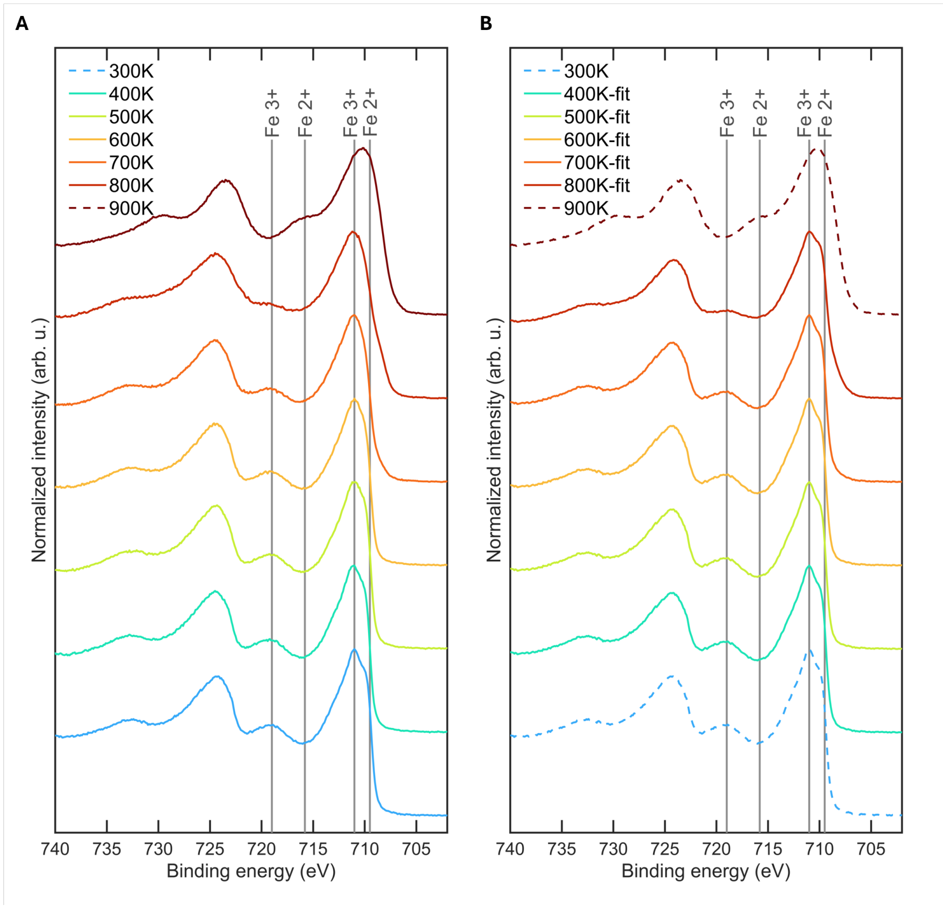

Figure S9: **Linear combination fitting of Fe 2p core-level spectra under reducing conditions.** A) Experimental Fe 2p spectra acquired using Al Kα excitation from 300 K to 900 K in 0.05 mbar H_2. B) Corresponding fitted spectra obtained by linear combination of reference spectra recorded at 300 K (Fe_2O_3-rich) and 900 K (FeO-rich) from the same sample.

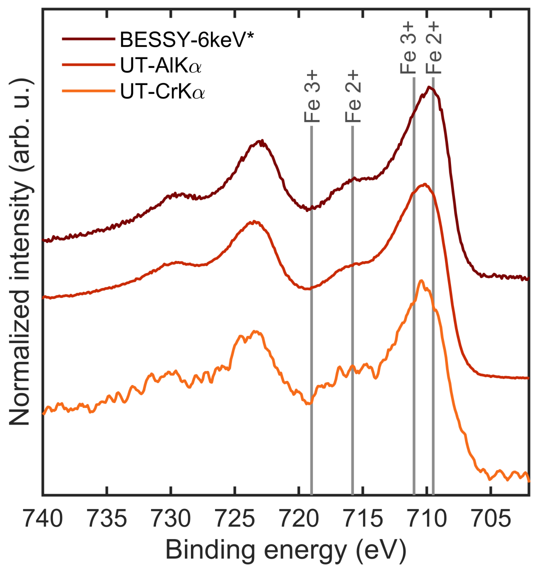

Figure S10: **Normalised XP core level spectra of Fe 2p in reducing condition at 900 K from Bessy synchrotron (*25*) and from our laboratory-based tool.** Total acquisition times: 30 minutes for synchrotron-based measurements, 15 minutes using the laboratory-based measurements with Al Kα excitation, and 38 minutes with Cr Kα excitation.

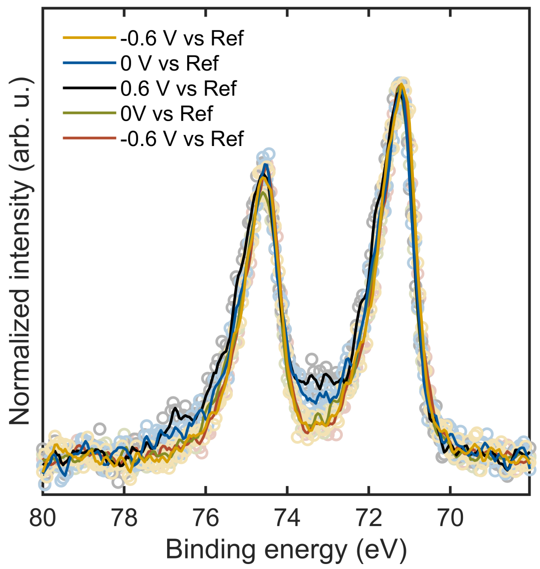

Figure S11: **XP spectra of the Pt 4f region excited with Al Kα photons during an experimental run on a 50 nm Pt thin film w orking e lectrode.** R ecorded c ore-level S pectra o f Pt 4 f a t d ifferent po tentials us ing Al Kα ex citation. These spectra were recorded using step size 0.05 eV, dwell time 1 s, pass energy 20 eV and 3 scans per potential. Open points represent the raw data. The solid lines show smoothened data with a quadratic Savitzky-Golay filter over 8 data points.

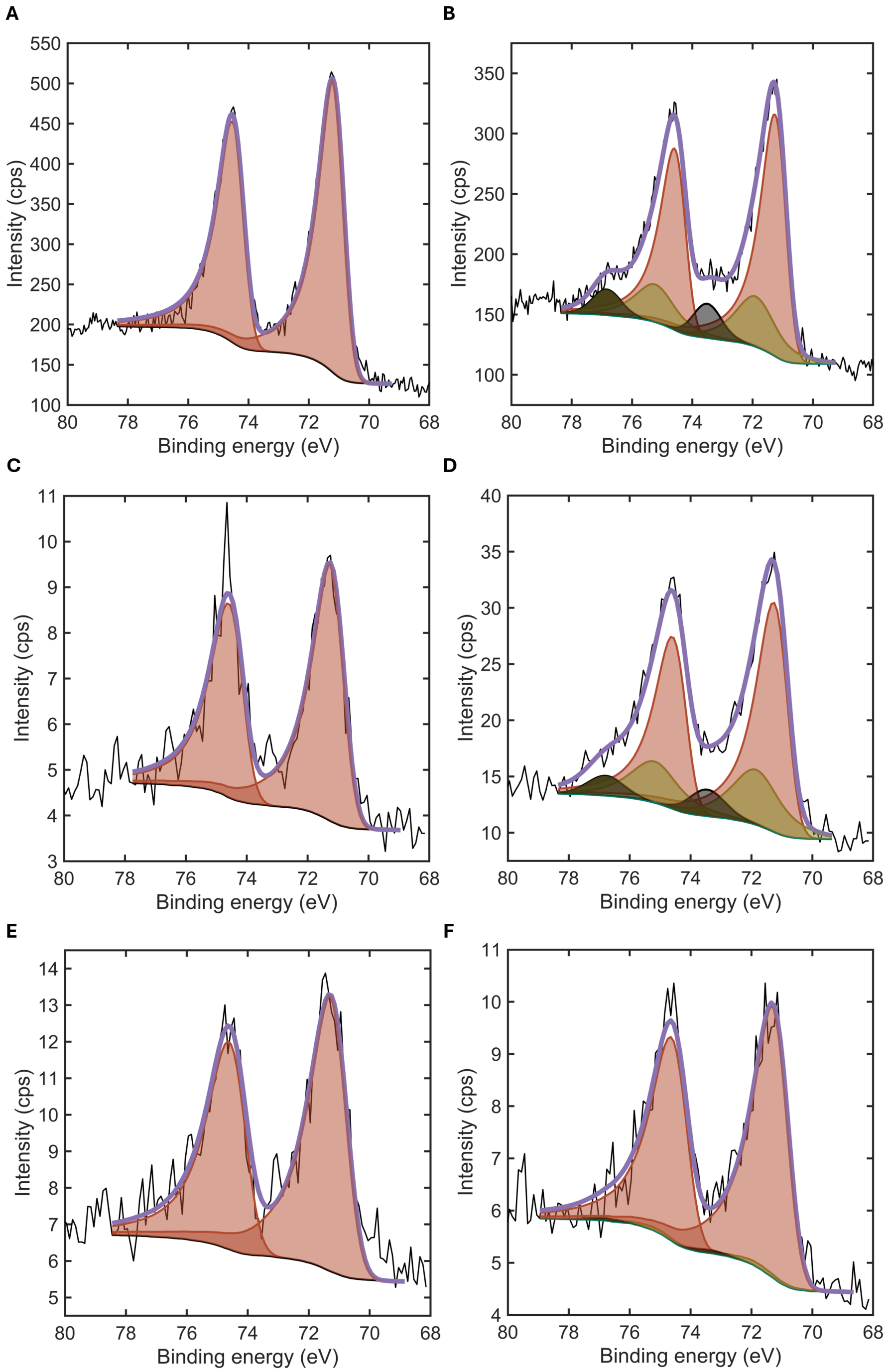

Figure S12: **XPS peak fitting of Pt 4f at -0.6 V and +0.6 V vs Ref using different X-ray excitations.** A, B) Al Kα. C, D) Ag Lα. E, F) Cr Kα. CasaXPS is used for peak fitting. First the Pt(0) only was fitted to the fully reduced sample at -0.6 V vs Ref, for the Al Kα excitation, which has the best signal-to-noise data quality. The best fit was used to determine constraints for the fitting of the Pt(0) component in the oxidized state, measured at 0.6 V vs Ref. Using only this metallic peak did not give a good fit, therefore, Pt-OH and Pt(II) where added to the fit as used by reference (*61*). Once a good fit was obtained, the peak model was applied for all spectra. At -0.6 V, i.e. in the reduced case, the fitting resulted in only metallic Pt(0) contribution. Therefore, only the metallic Pt(0) was fitted to the spectra for all excitations measured at -0.6 V vs Ref. Since the excitations have different FWHMs, only this restriction was changed between different excitations. The final fitting constraints can be found in table S1.

Table S1: **Fitting parameters for XP spectra of the Pt 4*f*.** Peak position, FWHM and the relative intensity for each peak for the three excitation energies. The spin orbit splitting was fixed to 3.33 eV and the $\frac{7/2}{5/2}$ peak ratio is 4:3.

	Position (eV)	FWHM (eV)
Al Kα source		
Pt(0)	71.01 ± 0.02	0.8 ± 0.5
Pt-OH	Pt(0) + 0.9	1.6 ± 0.5
Pt(II)	Pt(0) + 2.5	1.1 ± 0.5
Ag Lα source		
Pt(0)	70.95 ± 0.02	1.0 ± 0.5
Pt-OH	Pt(0) + 0.9	1.9 ± 0.5
Pt(II)	Pt(0) + 2.5	1.4 ± 0.5
Cr Kα source		
Pt(0)	70.94 ± 0.02	1.2 ± 0.5
Pt-OH	Pt(0) + 0.9	1.9 ± 0.5
Pt(II)	Pt(0) + 2.5	1.3 ± 0.5

Other Supplementary Materials for this manuscript

Movie S1: Real-time camera view of the switching of the tri-color source. Starting at Cr Kα (0 min), switching to Al Kα (1:10 min), to Ag Lα (2:45 min), and to Cr Kα (4:00 min) excitation lines on a phosphorous screen. The red elipse shows the expected beam location; the egdes of the analyzer cone and its shadow on the sample are also indicated. The bright white spot indicates the X-ray beam.
